# Supplementary material for: Effects of different fertilization modes on rice yield and quality under a rice-crab culture system
Source: PLoS One. 2020 Mar 25;15(3):e0230600. doi: 10.1371/journal.pone.0230600 (PMC7094854; doi:10.1371/journal.pone.0230600)
Supplement: Attachment — Submitted filename: PONE-D-20-01018_reviewer.docx [file pone.0230600.s001.docx]

**PLOS ONE**

3

4	Effect~~s~~ of different fertilization modes on rice yield

5	and quality under a rice-crab culture system

7 Wanning Zhao1¶, Hanling Liang1¶, Yu Fu1¶, Yubo Liu1¶, Chen Yang1¶, Tian Zhang1¶,

8 Tianyu Wang1¶, Liyan Rong1¶, Shuang Zhang1¶, Zhaoxia Wu1*, Wentao Sun2*

1 College of Food, Shenyang Agricultural University, Liaoning Province, Shenyang

2 110000, PR China

13 2	Institute of Plant Nutrition and Environmental Resources, Liaoning Academy of

4 Agricultural Sciences, Liaoning Province, Shenyang, 110161, PR China

17 ¶These authors contributed equally to this work.

8 *wuzxsau@163.com(Wu Z X); wentaosw@163.com(Sun W T)

19 Abstract

2

20 Rice-crab culture is the characteristic rice ecological breeding model used in the

21 Panjin area of Liaohe River Basin, China, and it can improve the ecological

22 environment and create increased economic benefits. From a food perspective, both

23 rice yield and quality, which are closely related to the fertilization mode, should be

24 considered. However, the effect of different fertilization modes on rice quality has not

25 been comprehensively investigated in this co-culture system. In this study, three local

26 optional fertilization modes, namely traditional mode FP1, one-time application of

7 mixed organic and inorganic fertilizer FP2, and one-time application of inorganic

28 fertilizer OPT, were evaluated for both rice yield and quality. In the rice-crab culture

9 system, FP2 used fewer fertilizers and had a lower economic cost, and the yield was

30 only slightly less than that of OPT but there was no statistical difference. FP2 elicited

31 the best appearance quality and better cooking and eating quality among all treatment

32 modes. ~~FP1, FP2, and OPT~~ significantly increased the protein content in rice and

33 decreased the amylose content, with . FP2 eliciting the lowest amount of protein

34 increase and amylose reduction. There was no significant change in crude fat and

35 starch content. Therefore, the FP2 fertilization mode was the best choice, as it

36 significantly improved rice yield and increased rice quality under the rice-crab culture

37 system at a relatively low cost.

38 Introduction

3

39 Rice (Oryza sativa L.) is one of the three major food crops worldwide. Approximately

40 50% of the world’s population uses rice as a staple food. Rice production in China

41 accounts for 30% of the world’s total production, ranking first worldwide. China has a

42 long history of rice cultivation and has formed a characteristic cultivation model that

43 conforms to the geographical environment [1]. The Liaohe River Basin is rich in

44 Chinese mitten crabs and is one of the three major crab producing areas in China.

45 Panjin City is located in the southern part of the alluvial plain of the Liaohe River

46 Basin. It is widely planted with rice and is one of the main rice-producing areas in the

47 Liaohe River Basin [2]. Since the last century, the Panjin area has gradually

48 developed a special cultivation mode of rice and crab culture, which has become an

49 important ecological agricultural model in the Liaohe River Basin. The organic

50 rice-crab culture not only increases rice yield, but also increases soil organic carbon

51 content, effectively improving the quantity and composition of soil carbohydrates

52 [3,4] . Rice and crab co-culture can control and reduce pests and weeds and decreases

3 the use of fertilizers and pesticides [5]. This co-culture also increases crab production

54 [6]. This co-culture system not only improves the ecological environment, but also

55 produces greater economic benefits. However, previous studies have focused on rice

56 yield under the rice-crab culture system and research on rice quality is rare [7].

57 Rice quality comprises milling quality, appearance, flavor, and eating and

58 nutritional quality, which are affected by many factors such as heredity and the

59 environment [8,9]. The application of fertilizer plays a crucial role in improving rice

60 yield and is also a key factor affecting rice quality [10]. Studies have shown that

4

61 different fertilizer types, as well as fertilization modes and rates directly affect rice

62 yield and quality [11-13]. The rice-crab culture system is no exception. The Panjin

63 area is located in the Liaohe River Basin. Local farmers have accumulated more than

64 10 years of experience in the fertilization technology of the rice-crab culture system

65 [14]. There are currently three representative fertilization modes: FP1 is the traditional

66 fertilization mode, with phosphorus and potassium fertilizers as the base fertilizer,

67 nitrogen fertilizer application rate as the base application, the tillering and heading

8 stages are 6:3:1. The FP2 mode utilizes a one-time application of a mixture of organic

69 and inorganic fertilizer. The OPT mode uses a one-time application of the

70 recommended inorganic fertilizer based on the balanced fertilization technique. To

71 date, no studies have examined the effects of these three different fertilization modes

72 on rice yield and quality in the rice-crab culture system in the Panjin area. Therefore,

3 the present study used rice variety Yanfeng 47 as the experimental material to study

74 the difference in yield, milling, appearance, cooking and eating, and nutritional

75 quality of rice in the rice-crab culture system. Based on the comprehensive

76 measurement of rice yield and quality under the above three fertilization modes, the

77 fertilization mode and amount suitable for producing rice under the rice-crab culture78 system in the Panjin area were determined.

79 Materials and methods

80 Site description and plant materials

81	The test area of the present study was located at Hangcheng Farm (122°14′17″ N,

82 41°9′31″ E) of Liaoning Province, China (Banba Town, Panshan County). The tested

5

3 soil was saline soil. The soil parameters were as follows: pH 8.20, organic matter 22.6

84 g/kg, total nitrogen 1.42 g/kg, available nitrogen 105.2 mg/kg, effective phosphorus

85 21.6 mg/kg, available potassium 164.2 mg/kg, and bulk density was 1.39 g/cm3. The

86 sample used was the main rice variety Yanfeng 47 from the coastal rice-growing area

87 of Liaoning. Chinese mitten crabs were symbiotic with this rice. The rice was

transplanted on May 25, 2018 and harvested on October 8. The crab juveniles were

9 admitted to the farm on May 28 and harvested on September 30. Each planting area

90 was 75 m2, the plot was 25 m long and 3 m wide, and the planting distance × plant

1 spacing was 9 inches × 5 inches. Plastic spaces were built between the communities

92 and the peripheral network of the community was used to prevent crabs from fleeing.

93 Each experiment was repeated three times.

94 Experimental design

95	The control and fertilization modes used in this experiment were conducted as

96 follows: 1) CK: control, no fertilization. 2) FP1: traditional fertilization mode, 100%

97 inorganic fertilizer, and total fertilization amount of 540 kg hm-2, of which the ratio of

98 nitrogen, phosphorus, and potassium was 20:7:9. Phosphorus and potassium fertilizers

99 were applied together as the base fertilizers, and 60% of the nitrogen fertilizers were

applied as base fertilizers, 30% were applied at the tillering stage, and 10% were

101 applied at the heading stage. 3) FP2: One-time application of organic-inorganic

102 compound fertilizer, with a total fertilization amount of 379.5 kg hm-2, of which the

03 ratio of nitrogen, phosphorus, and potassium was 14:5:6.3. 4) OPT: One-time

104 application of inorganic fertilizer, with a total fertilization amount of 465 kg hm-2, of

6

05 which the ratio of nitrogen, phosphorus, and potassium was 18:7:6. This was

106 considered by the local farmers to be the best amount of fertilizer to apply.

107 Experimental methods

08	Rice was harvested at maturity. After drying to a moisture content of 14%, the

9 rice was stored at room temperature (15-30℃ ) and then subjected to quality

110 measurements.

Milling quality: was based on the GB/T 17891-1999 (1999) standard to

112 determine the brown, milled, and head rice rates. The rice was shelled two times using

113 a rice sheller (FC2K, Yamamoto, Japan) and rice machine (VP-32T, Yamamoto, Japan)

114 to obtain brown rice and milled rice, respectively, and weighed separately.

115	Appearance quality: chalky kernel rate and chalkiness were measured by image

6 processing measurement in GB 1354 using an ES-1000 appearance quality detector117 (Shizuoka, Japan).

118	Cooking and eating quality: the cooked rice soup was cooled to room

9 temperature and the pH of the rice was measured with a PB-10 pH meter (German

120 Sartorius Group). The expansion ratio was measured by the drainage method where

121 2.5 g of rice was placed in 50 mL of water and the elevated volume was measured.

122 After cooking and draining, the rice was placed in 50 mL of water and the elevated

123 volume was measured. The water absorption rate was measured by weighing 2.5 g of

124 rice and 50 mL of water, cooking, draining, and then reweighing the rice with a filter

paper (M after cooking). Water absorption (%) = M after cooking - 2.5/2.5 × 100. The cooking

126 time was determined by pressing the cooked rice grains in a hand and determining

7

127 when there was no hard heart in the middle, and the time was recorded. The taste

128 value score was measured by pressing the cooked rice into a rice cake and measuring

29 the taste with a STA1B taste scoring apparatus (Satake, Japan). The texture

130 characteristics (e.g., hardness, viscosity, and elasticity) were measured by pressing the

131 cooked rice into a rice cake and measuring the textural characteristics of the rice cake

132 using a CT34500 texture analyzer (Brookfield Engineering Laboratories). The probe

133 (TA43 spherical probe) was set at 30 mm above the platform, the test speed was 2.5

134 mm/s, the test speed was 5 mm/s after the test, the trigger point load was 0.05 N, the

135 target value was 65%, and the cycle was repeated twice. Starch pasting characteristics

136 were measured using a rapid viscosity analyzer. A total of 3 g of milled rice flour was

137 sampled, 25 mL of distilled water was added, and this mixture was placed into an

138 Rapid viscosity analyzer (RVA-4) (Newport Scientific, Australia) for determine the

139 starch pasting characteristics.

140	Nutritional quality: protein and amylose content were determined with a fixed

141 grating near-infrared automatic analyzer (DA7200, Perten, Sweden). The brown rice

142 samples were tiled and filled the sample trays, and the brown rice mode was selected

143 for automatic scanning. The crude fat was determined by Soxhlet extraction using

44 GB5009.6-2016, with a sample of 1.00 g of brown rice flour. The starch content was

145 determined by pretreatment of the rice sample using the acid hydrolysis method from

the GB 5009.9-2016 standard, then 10 mL of the sample solution was added to 490

147 mL of distilled water, 0.5 mL of phenol, and 2.5 mL of concentrated sulfuric acid, and

148 the mixture was placed in a test tube. After cooling, the absorbance was measured at

8

149 490 nm using an ultraviolet-visible spectrophotometer (UV762 Shanghai Dingke

150 Scientific Instrument Co., Ltd.).

151 Statistical analysis

152	The data in all tables are the average values of three repeated observations. Data

53 was sorted by Excel 2010 and the significant and multivariate correlation analyses

154 were performed using the SPSS 25 software program.

155 Results

156 ~~Rice yields of the rice-crab culture system~~

57	The rice yields are shown in Table 1. Compared with CK, FP1, FP2, and OPT

158 showed significant increases in rice yield, increasing by 52.0%, 64.0%, and 75.5%,

159 respectively. However, there was no significant difference in the rice yield obtained

160 among the three different fertilization modes. The highest yield was obtained by OPT,

161 which was 10 040 kg·hm-2.

62 Table 1. Effects of different fertilization modes on rice yield under the rice-crab culture

9

163 system

Treatment	Fertilization rate (kg·hm-2)	Nitrogen rate (kg·hm-2)	Yield (kg·hm-2)

CK	0	0	5720 ± 1869b

FP1	540	300	8693 ± 107a

FP2	379.5	210	9377 ± 821a

OPT	465	270	10 040 ± 767a

64 CK: no fertilization; FP1: conventional fertilization, inorganic fertilizer; FP2: one-time

165 fertilization, organic-inorganic compound fertilizer; OPT: one-time fertilization, inorganic

166 fertilizer. Different letters indicate statistical significance at the P = 0.05 level.

167 2.Rice quality characteristics ~~of the rice-crab culture system~~

168 2.1.Rice milling quality and appearance quality

169	The rice milling quality and appearance quality results are shown in Table 2. The

70 brown rice and head rice rates of FP1 increased significantly by 5.2% and 22.8%,

171 respectively, compared with CK. The other two fertilization modes were not

172 significantly different from CK. FP2 had the highest milled rice rate; however, there

173 was no significant difference in the milled rice rates among the different fertilization

174 modes. For the rice appearance quality, the chalky kernel rate of rice after the

175 different fertilization treatments decreased significantly compared with ~~that of~~ CK.

6 The three fertilization modes of FP1, FP2, and OPT decreased by 15.5%, 15.7%, and

77 11.8% respectively. There was no significant difference between the chalkiness. FP2

178 had the lowest chalky kernel rate and chalkiness.

179 Table 2. Rice milling quality and appearance quality under the rice-crab culture system

10

Milling quality (%)	Appearance quality (%)

Treatment

Brown rice	rate

Milled rice	rate

Head rice	rate Chalky kernel	rate

Chalkiness

CK	78.44±1.11b 78.61±3.26a 52.11±5.05b	44.73±5.13a	12.62±2.52a

FP1	82.52±2.96a 75.21±4.90a 63.98±6.28a	37.78±7.65b	12.2±2.84a

FP2	78.60±0.91b 79.54±1.82a 58.52±4.94ab	36.99±7.04b	11.02±3.04a

OPT	78.17±1.06b 78.13±2.34a 61.58±6.72ab	39.47±2.25b	13.29±0.90a

80 CK: no fertilization; FP1: conventional fertilization, inorganic fertilizer; FP2: one-time

181 fertilization, organic-inorganic compound fertilizer; OPT: one-time fertilization, inorganic

182 fertilizer. Different letters indicate statistical significance at the P = 0.05 level.

183 2.3.Rice cooking and eating quality

84	The rice cooking and eating quality results are shown in Figs 1-3. There was no

185 significant difference in the cooking quality and texture characteristics of rice treated

86 with different fertilization (Figs 1 and 3). Compared with CK, the FP1 treatment

187 significantly reduced the appearance score of rice (Fig 2A). The three different

188 fertilization modes increased the hardness score of rice, and reduced the viscosity

189 score and balance score, with significant differences among FP1, OPT, and CK (Figs

190 2B–D). However, there was no significant difference between FP2 and CK (Figs 1–3).

191 Therefore, the FP2 fertilization mode did not cause significant changes in the cooking

92 and eating quality of rice, whereas the FP1 and OPT fertilization modes significantly

193 reduced the eating quality.

194

95	Fig 1. Rice cooking quality under the rice-crab culture system. CK: no fertilization; FP1:

11

196 conventional fertilization, inorganic fertilizer; FP2: one-time fertilization, organic-inorganic

197 compound fertilizer; OPT: one-time fertilization, inorganic fertilizer. Different letters indicate

198 statistical significance at the P = 0.05 level.

99 Fig 2. Rice eating quality under the rice-crab culture system. CK: no fertilization; FP1:

00 conventional fertilization, inorganic fertilizer; FP2: one-time fertilization, organic-inorganic

201 compound fertilizer; OPT: one-time fertilization, inorganic fertilizer. Different letters indicate

202 statistical significance at the P = 0.05 leve

203	Fig 3. Rice texture characteristics under the rice-crab culture system. CK: no fertilization;

12

04 FP1: conventional fertilization, inorganic fertilizer; FP2: one-time fertilization, organic-inorganic

205 compound fertilizer; OPT: one-time fertilization, inorganic fertilizer. Different letters indicate

206 statistical significance at the P = 0.05 level.

207	The rice starch pasting properties results are shown in Table 3. There was no

208 significant difference b~~etwee~~n the breakdown, setback, peak time, and pasting

209 temperature of the rice in any of the different fertilization modes. The peak, trough,

210 and final viscosities of the four treatments had consistent results, with the FP1

211 treatment significantly lower than that of the CK, and was decreased by 18.0%,

17.28%, and 13.9%, respectively. Therefore, the eating quality of the rice in the FP1

13 mode was poor. However, there was no significant difference among FP2, OPT, and

214 CK.

215 Table 3. Rice starch pasting properties under the rice-crab culture system

Treatment

Peak viscosity

(RVU)

Trough viscosity

(RVU)

Breakdown

(RVU)

Final viscosity

(RVU)

Setback(RVU)

Peak time

(min)

Pastingtemperature (℃)

CK	3207±424.58a 2089.33±161.97a 1117.66±283.86a 3102±270.25a 1012.66±120.14a 6.24±0.28a 72.98±0.49a

FP1	2631±48b	1728.33±56.5b	902.66±8.5a	2672±60.63b	943.66±29.93a 6.33±0a	72.98±0.44a

FP2 2827.33±123.45ab 1913±104.93ab 914.33±80.25a 2844.33±99.52ab 931.33±19.73a 6.35±0.07a 73.21±0.49a

OPT 2890.33±189.89ab 1917±156.61ab 973.33±67.57a 2877.33±185.13ab 960.33±45.54a 6.29±0.03a 72.65±0.05a

6 CK: no fertilization; FP1: conventional fertilization, inorganic fertilizer; FP2: one-time

217 fertilization, organic-inorganic compound fertilizer; OPT: one-time fertilization, inorganic

218 fertilizer. Different letters indicate statistical significance at the P = 0.05 level.

2192.4 Rice nutritional quality

220	The rice nutritional quality results are shown in Fig 4. There were no significant

13

221 differences in crude fat and starch content among the three fertilization modes and CK

(Figs 4B and C). Compared with CK, FP1, FP2, and OPT had a significantly higher

223 protein content of 45.7%, 19.8%, and 43.3%, respectively, and FP1 and OPT were

significantly higher than FP2 (Fig 4A). The three fertilization modes significantly

5 reduced the amylose content by 3.0%, 1.4%, and 2.6%, respectively, and FP1 and

6 OPT were significantly lower than FP2 (Fig 4D). There was no difference in starch

227 content between rice in the different fertilization modes; however, there was a

228 significant difference in amylose content, indicating that different fertilization modes

229 may affect the type and quantity of starch synthesis. The protein content of the rice in

30 the four modes showed a trend of FP1 &gt; OPT &gt; FP2 &gt; CK, whereas the amylose trend

231 was the opposite.

232 Fig 4. Rice nutritional quality under the rice-crab culture system. CK: no fertilization; FP1:

33 conventional fertilization, inorganic fertilizer; FP2: one-time fertilization, organic-inorganic

234 compound fertilizer; OPT: one-time fertilization, inorganic fertilizer. Different letters indicate

235 statistical significance at the P = 0.05 level.

236.2.5. Correlation between rice quality traits

237	The correlation analysis results are shown in Table 4. The head rice rate was

238 significantly positively correlated with protein content and hardness score; however, it

239 was negatively correlated with appearance, viscosity, balance, and taste scores, and

240 other cooking and eating quality and amylose content. The chalkiness was

241 significantly positively correlated with the water absorption rate. There was a

242 significant and negative correlation between protein content and appearance, viscosity,

14

243 balance, and taste scores, and other cooking and eating quality, and a significant

244 positive correlation with hardness score. Amylose was the opposite of protein and

245 there was a significant negative correlation between ~~the two~~.

246 Table 4. Correlation between rice quality traits under the rice-crab culture system

Head rice

rate

Chalkiness pH Expansion

Cooking

time

Hardness

score

Breakdow

n

viscosity

content

1

-0.388 0.217 -0.811 1

Chalkiness -0.137 -0.257 0.032 0.506 1

pH -0.295 0.644 -0.332 -0.280 -0.904 1

15

Brownrice rate

Milled rice

rate

Chalky

kernel rate

ratio

Waterabsorption

rate

Appearanc

e score

Viscosity

score

Balance

score

Taste score Hardness Viscosity Elastic Peak

Troughviscosity

Finalviscosity

Setback Peak time Pasting

temperatur

e

Proteincontent

Fat content Starch

Brownrice rate

Milled rice

rate

-0.921 1

Head rice

rate

0.608 -0.642 1

Chalky

kernel rate

Expansion

ratio

-0.779 0.498 -0.140 0.263 0.624 -0.287 1

Water

absorption

-0.336 -0.058 -0.027 0.490 .975* -0.800 0.782 1

Cooking

time

0.457 -0.732 0.744 -0.222 0.677 -0.873 0.202 0.589 1

Appearanc

e score

-0.691 0.743 -.990* 0.739 -0.093 0.422 0.197 -0.006 -0.794 1

Hardness

score

0.681 -0.750 .986* -0.713 0.137 -0.460 -0.168 0.049 0.821 -.999** 1

Viscosity

score

-0.625 0.697 -.993** 0.738 -0.136 0.438 0.110 -0.065 -0.814 .996** -.997** 1

Balancescore

-0.659 0.726 -.991** 0.730 -0.128 0.443 0.149 -0.047 -0.813 .999** -.999** .999** 1

Taste score -0.662 0.725 -.992** 0.735 -0.119 0.436 0.155 -0.038 -0.808 .999** -.999** .999** 1.000** 1

Hardness 0.517 -0.779 0.751 -0.225 0.652 -0.872 0.134 0.549 .997** -0.808 0.833 -0.821 -0.823 -0.819 1

Viscosity -0.019 -0.221 0.723 -0.447 0.509 -0.527 0.580 0.560 0.788 -0.683 0.703 -0.745 -0.718 -0.713 0.748 1

Elastic -0.787 0.483 -0.345 0.522 0.714 -0.356 .960* 0.846 0.137 0.372 -0.338 0.295 0.327 0.334 0.076 0.396 1

Peakviscosity

16

-0.717 0.611 -0.934 0.906 0.304 0.049 0.436 0.378 -0.494 0.920 -0.902 0.899 0.905 0.909 -0.517 -0.452 0.638 1

Troughviscosity

-0.821 0.753 -0.935 0.806 0.187 0.201 0.482 0.297 -0.593 0.948 -0.935 0.920 0.932 0.935 -0.623 -0.433 0.647 .981* 1

Breakdow

n

-0.510 0.354 -0.867 .990* 0.457 -0.181 0.335 0.471 -0.312 0.813 -0.788 0.805 0.801 0.806 -0.322 -0.449 0.580 .957* 0.882 1

Finalviscosity

-0.757 0.663 -0.938 0.874 0.266 0.101 0.456 0.353 -0.529 0.933 -0.917 0.909 0.918 0.921 -0.555 -0.445 0.646 .998** .992** 0.935 1

Setback -0.357 0.172 -0.779 .999** 0.543 -0.329 0.265 0.519 -0.172 0.703 -0.675 0.702 0.693 0.698 -0.174 -0.417 0.524 0.885 0.778 .982* 0.851 1

Peak time 0.409 -0.159 0.685 -.972* -0.690 0.455 -0.428 -0.682 0.023 -0.615 0.581 -0.599 -0.596 -0.602 0.035 0.224 -0.662 -0.859 -0.752 -.957* -0.826 -.979* 1

Pastingtemperatur

0.158 0.231 -0.270 -0.226 -0.945 0.859 -0.723 -.955* -0.782 0.296 -0.337 0.355 0.336 0.328 -0.745 -0.760 -0.721 -0.089 -0.014 -0.193 -0.065 -0.263 0.448 1

Proteincontent

0.536 -0.648 .980* -0.698 0.229 -0.491 0.015 0.174 0.853 -.977* .983* -.992** -.986* -.985* 0.852 0.822 -0.172 -0.845 -0.863 -0.758 -0.854 -0.661 0.536 -0.457 1

Fat

-0.745 0.509 0.019 0.033 0.488 -0.180 .971* 0.662 0.213 0.063 -0.040 -0.028 0.015 0.021 0.142 0.679 0.867 0.254 0.333 0.116 0.285 0.032 -0.201 -0.657 0.147 1

content

Starchcontent

-0.080 -0.238 0.575 -0.188 0.726 -0.702 0.678 0.764 0.835 -0.561 0.591 -0.626 -0.600 -0.594 0.794 .961* 0.560 -0.250 -0.269 -0.204 -0.256 -0.154 -0.049 -0.910 0.718 0.713 1

Amylose

content

-0.583 0.665 -.992** 0.739 -0.156 0.440 0.060 -0.094 -0.819 .990* -.992** .999** .995** .995** -0.823 -0.778 0.251 0.886 0.901 0.800 0.894 0.703 -0.591 0.384 -.997** -0.080 -0.659 1

247 *, significant at the P = 0.05 level; **, significant at the P = 0.01 level.

248 Discussion

17

249 ~~Effects of different fertilization modes on rice yield under~~

~~250 the rice-crab culture system~~

251	Rice yield is the result of a combination of rice genetics, various environmental

252 factors, fertilization modes, and nitrogen application rates; thus, the factors affecting

253 yield are diverse and complex [15]. The results of ~~the effects of the rice-crab culture~~

~~254 system~~ on rice yield obtained from previous studies differ to each other. Some

255 ~~scholars~~ believed that the rice-crab culture system would increase rice yield [16,17].

256 This might be due to the additional nutrients from crab feces, crab movements

257 accelerating the release of accumulated nutrients, and crab feeding on aquatic plants

258 contributing to nutrient recycling and decreasing loss of N [14]. However, other

259 scholars believed that the presence of crabs had no significant effect on rice yield [18].

260 In the present study, the rice yield in the crab-free system that had the same variety

261 and planting environment and the same fertilization mode and amount as that found in

262 FP1 was measured. The yield of rice in the crab-free system was 11 017 kg·hm-2 (data

263 not shown), which was slightly higher than that in the rice-crab culture system;

264 however, there was no significant difference in the yield difference among the three

265 fertilization modes. This might be due to the long-term maintenance of the deep-water

266 layer, which is not conducive to rice root growth and tillering, or because the crabs

267 crawl in the field, causing damage to the rice tillers and decreasing the number of

268 effective panicles [19].

269	In the rice-crab culture system, the main factors affecting rice yield are the

270 fertilization mode and nitrogen application rate. After fertilization, the rice yield

18

271 increased significantly. However, this increase was not proportional to the amount of

272 nitrogen applied. The fertilizer application rate of the traditional fertilization mode,

273 FP1, was too ~~large~~, which not only reduces rice yield but also causes soil pollution. In

274 the traditional fertilization mode, the application amount of nitrogen, phosphorus, and

275 potassium was unreasonable and the application of the base fertilizer was emphasized;

276 therefore, the early stage of rice growth was too strong, the effective tiller was

277 decreased, and the ineffective tiller increased, which was the possible cause of the

8 decline in yield [20]. Jia et al. [21] also studied the Yanfeng 47 rice variety and found

279 that the application of nitrogen fertilizer increased the number of tillers for Yanfeng

280 47, thus increasing the yield. However, excessive application of nitrogen fertilizer

281 leads to delayed or immature rice growth. Therefore, reasonable control of the

282 application rate of nitrogen fertilizer creates high rice yield. The nitrogen application

83 rate of FP2 was 22.2% lower than that of OPT and yet the rice yield was only

284 decreased by 6.6% and there was no significant difference from that of OPT. This

85 might be due to the application of organic fertilizer to FP2 for increasing the rice yield

86 [22]. The combination of organic and inorganic fertilizers can promote nutrient

287 synchronization and nitrogen use efficiency and reduce nutrient loss by converting

88 inorganic nitrogen into organic forms [23]. An et al. [24] found that when the external

289 environmental factors were good, the appropriate application of organic fertilizer

290 increased rice yield under the rice-crab culture system, which was close to or even

91 higher than simply planting rice. Walia et al. [25] also showed that the combination of

292 organic and chemical fertilizers increased the 1000-grain weight, protein content, and

19

293 quality parameters of rice compared with chemical fertilizer alone. In the present

94 study, the FP2 mode showed lower economic costs and higher rice yield. Therefore, in

95 terms of yield, the FP2 application of organic-inorganic compound fertilizer was more

296 suitable for rice production in the rice-crab culture system.

297 Effects of different fertilization modes on rice quality under298 the rice-crab culture system

299	As the main measure of rice quality, the head rice rate reflects the milling quality

300 and commodity value of rice milling [26]. With an increase in the nitrogen application

301 rate, the brown rice and head rice rates of FP1 were significantly improved.

302 Correlation between rice quality showed that the head rice rate was significantly

303 positively correlated with the protein content and hardness score, with correlation

304 coefficients of 0.980 and 0.986, respectively; however, it was negatively correlated

305 with the appearance, viscosity, balance, and taste scores, and other cooking and eating

306 quality and amylose content. Therefore, the better the quality of the milling, the worse

307 the cooking and eating quality of the rice. This was mainly due to the increase of

308 protein content and decrease of amylose content in rice, as well as the increase of rice

309 hardness and the ability of rice to resist grinding damage. However, palatability was

310 reduced.

311	Chalkiness is a white opaque area produced by the loose accumulation of starch

312 granules in rice grains and is an unfavorable feature of the appearance quality of rice

313 grains [27]. Chalkiness also reduces the milling and cooking and eating quality of rice

314 [28]. Therefore, rice production should aim to reduce the chalkiness of rice. The

20

315 formation of rice chalkiness is significantly affected by rice varieties and

316 environmental factors such as nitrogen application and temperature [29]. In the

317 present study, the application of nitrogen fertilizer significantly reduced the chalky

318 kernel rate of rice. This was due to the application of nitrogen fertilizer to cause

319 protein accumulation in rice, resulting in more space occupied by protein bodies,

20 dense starch particles, and improved appearance quality [30]. Qiao et al. [31] also

321 showed that nitrogen fertilizer had a good effect on decreasing the chalky kernel rate.

322 However, with a further increase in the nitrogen level, the total amount of spikelets

323 increased, and the grain filling period was enlarged, resulting in lower grain filling

324 rate, poor grain filling, and increase in false grains. In this case, the arrangement of

325 the starch granules was loose, resulting in chalkiness of the rice [32]. Chalkiness was

326 significantly positively correlated with the water absorption rate in the present study

327 (R2 = 0.975). The higher whiteness indicated that the starch granules in the rice were

328 loosely arranged and the voids were large. The starch easily swelled during cooking;

329 therefore, the water absorption rate was high.

330	In terms of cooking and eating quality, rice produced in the FP1 fertilization

331 mode was the worst and FP2 was the best. Starch is the main component in rice and

332 starch pasting significantly affects the texture of rice [33]. The RVA results showed

333 that the peak, trough, and final viscosities of FP1 were significantly lower than that of

334 CK; however, there was no significant difference between FP2 and CK. According to

335 the correlation results, the rice taste score was positively correlated with the starch

336 pasting viscosity, although the correlation was not significant. However, it was

21

337 enough to prove that the cooking quality of the FP1 rice was poor.

8	Protein content can significantly affect the cooking and eating quality of rice [34].

339 The results of the present study indicated that the protein content increased as the

340 nitrogen application rate increased in the rice-crab culture system. There was a

341 significant negative correlation between protein content and appearance, viscosity,

342 balance, and taste scores, and other cooking taste quality (R2 = -0.977, -0.992, -0.986,

343 -0.985, respectively) and a significant positive correlation with hardness score (R2 =

44 0.983). This was in agreeance with the results of the cooking and eating quality

345 measurements. Nitrogen application resulted in a significant increase in protein

346 content in rice and a decrease in cooking and eating quality. The lowest protein

347 content of FP2 had the highest taste score and the rice had the best cooking and eating

348 quality. This might be due to the network structure formed by the disulfide bond of

349 the protein to affect the starch pasting, making the rice texture harder and less viscous,

350 resulting in a poor taste quality of the rice [35]. Baxter et al. [36] showed that the

351 most abundant gluten in rice protein interacted directly with the starch granules,

352 blocking the entry of water molecules and resulting in slower water absorption of the

353 starch granules and lower water absorption rate. This effect was concentration

354 dependent and was consistent with the results of the present study. Protein content

355 was a key factor in terms of milling, appearance, and cooking and eating qualities.

356 However, the application rate of nitrogen in each mode was not the same and the

357 nitrogen content in the field was complicated towing to the residues of crab feed and

358 the excretion of crab manure. The mechanism of the effect on rice quality requires

22

359 further evaluation.

360	Starch includes two types of molecules: amylose and amylopectin. Most scholars

361 believe that the amylose and protein content in rice is the most important chemical

62 component determining the cooking and eating quality of rice [37]. The mechanism of

363 the effect of starch on rice texture must be further studied. In the present study,

364 amylose content decreased as the amount of nitrogen applied in the four fertilization

365 modes increased. This might be because the application of nitrogen fertilizer delayed

366 the accumulation of starch during the early stage of grain filling and significantly

367 reduced the amylose content. Rice containing lower amylose has better softness and

368 palatability and is preferred by consumers. However, rice with higher amylose content

69 tends to have a harder and less sticky texture after cooking [38]. This is due to the

370 amylose molecules limiting starch swelling and leaching during cooking by

371 entanglement and/or co-crystallization with amylopectin, thus affecting the viscosity

72 between cooked rice grains [39] and resulting in a harder texture, limited starch

373 leaching, reduced eating quality of rice [40-41]. Although the application of nitrogen

374 fertilizer reduced the amylose content and improved the cooking and eating quality,

375 excessive nitrogen application significantly increased the protein content and

376 ultimately reduced the cooking and eating quality of rice. This was consistent with the

377 results of the correlation analysis, where there was a significant positive correlation

378 between amylose and appearance, viscosity, balance, and taste scores, and other

379 cooking and eating quality (R2 = 0.990, 0.999, 0.995, 0.995, respectively), and

380 negatively correlated with hardness score (R2 = -0.992). In summary, reasonable

23

381 fertilization was the key to improving rice quality.

382 Conclusions

383	In the rice-crab culture system, the three fertilization modes significantly

384 increased the rice yield compared with the control, but there was no significant

385 difference in the yield under the three modes. FP1 treatment improved the milling

386 quality and FP2 treatment improved the appearance quality. ~~Amon~~g the three

387 treatments, FP2 treatment maintained good eating quality. From the perspective of

388 nutritional quality, the protein content of the FP2 treatment was reasonable, although

389 it was not the highest, but it contributed to the formation of a good comprehensive

390 quality of rice. In addition, FP2 treatment reduced the amount of nitrogen fertilizer

391 applied compared with traditional fertilization mode, the cost was reduced, and the

392 yield could be guaranteed. ~~It was the recommended fertilization mode~~ for the

393 rice-crab culture system in Panjin area .

394	The rice-crab culture model has been proven to improve the ecological

395 environment of rice fields and increase economic benefits. In the future, focus should

396 be ~~applied~~ to improv~~ing~~ modern rice cultivation management techniques and rice-crab

397 culture techniques and adopting a reasonable fertilization mode to further improve the

398 yield and quality of rice.

399 Acknowledgments

24

400	This work was supported by grants from the National Key Research and

401 Development Program of China (grant no. 2018YFD0200203) and Science and

402 Technology Innovation Talents Training Project of Liaoning Province (grant no.

3 XLYC1802044).

404 Author contributions

405 Conceptualization: Zhaoxia Wu, Wanning Zhao

406 Data curation: Wanning Zhao, Hanling Liang, Yu Fu, Yubo Liu, Chen Yang, Tian

407 Zhang, Tianyu Wang, Liyan Rong, Shuang Zhang

408 Formal analysis: Wanning Zhao

409 Funding acquisition: Zhaoxia Wu, Wentao Sun

410 Project administration: Zhaoxia Wu, Wentao Sun, Wanning Zhao, Hanling Liang

411 Writing - original draft: Wanning Zhao

412 Writing - review &amp; editing: Zhaoxia Wu, Wanning Zhao

413 References

25

414 1. Li MY, Chen YY, Yang Y, Meng GY, Zhou J. Research progress of high yield cultivation mode of rice. Agric

5	Sci Tech. 2017b; 18(8):1416-1421. http://d.old.wanfangdata.com.cn/Periodical/hnnykjtx-e201708014

416 2. Xu Q, Wang XL, Xiao B, Hu KL. Rice-crab coculture to sustain cleaner food production in Liaohe417	River Basin, China: An economic and environmental assessment. J Clean Prod. 2018; 208:188-198.

418	https://doi.org/10.1016/j.jclepro.2018.10.092

419 3. Liu MD, Hao XD, An H, Yan Y, Wang YJ. Effects of different rice-crab production modes on soil

humus characteristics in different aggregates. J Shenyang Agric Univ. 2012; 43(3):306-310. http://w**w**

421	w.en.cnki.com.cn/Article_en/CJFDTotal-SYNY201203009.htm

22 4. Yan Y, Liu MD, Yang D. Effect of different rice-crab coculture modes on soil carbohydrates. J Integr Agr.

2014; 13(3):641-647. https://doi.org/10.1016/S2095-3119(13)60722-4

424 5. Zheng HB, Huang H, Chen C, Fu ZQ, Xu HQ, Tan SD, et al. Traditional symbiotic farming techno425	logy in China promotes the sustainability of a flooded rice production system. Sustain Sci. 2017; 12:

426	155–161. https://link.springer.com/10.1007/s11625-016-0399-8

427 6. Chen Y, Niu SW, Zou XJ, Wang N, Xu JY. Effect of rice-crab co-culture on environment of rice field.

Liaoning Agric Sci. 2018; 3:30-34. http://www.cnki.com.cn/Article/CJFDTotal-LNNY201803011.htm

429 7. Meng SL, Hu GD, Li DD, Qiu LP, Song C, Fan LM, et al. Research progress of rice-fish integrated farming.

Agric Biotech. 2018; 7(4):129-135. http://en.cnki.com.cn/Article_en/CJFDTotal-AGBT201804034.htm

431 8. Cai YX, Liu CX, Wang W, Cai KZ. Differences in physicochemical properties of kernels of two ric432	e cultivars during grain formation. J Sci Food Agric. 2011; 91:1977–1983. https://www.ncbi.nlm.nih.g

33	ov/pubmed/21480275

434 9. Wang A. Migration and transformation of nitrogen in rice-crab culture systems. Shanghai Ocean University,

35	Shanghai. 2018. Available from: http://cdmd.cnki.com.cn/Article/CDMD-10264-1018848657.htm

436 10. Liu X, Li M, Guo P, Zhang ZX. Optimization of water and fertilizer coupling system based on rice grain

7	quality. Agr Water Manage. 2019; 221:34-46. https://doi.org/10.1016/j.agwat.2019.04.009

438 11. Hao HL, Wei YZ, Yang XE, Feng Y, Wu CY. Effects of different nitrogen fertilizer levels on Fe,

39	Mn, Cu and Zn concentrations in shoot and grain quality in rice (Oryza sativa). Rice Sci. 2007; 1**4**

(4):289-294. https://doi.org/10.1016/S1672-6308(08)60007-4

441 12. Liu QH, Ma H, Lin XQ, Zhou XB, Zhao QL. Effects of different types of fertilizers application on rice grain442	quality. Chil J Agr Res. 2018; 79(2):202-209.

443 13. Tang S, Zhang HX, Liu WZ, Zhi D, Zhou QY, Chen WZ, et al. Nitrogen fertilizer at heading stage effectively444	compensates for the deterioration of rice quality by affecting the starch-related properties under elevated

temperatures. Food Chem. 2019; 227:455-462. https://doi.org/10.1016/j.foodchem.2018.10.137

6 14. Wang A, Ma XZ, Xu J, Lu WQ. Methane and nitrous oxide emissions in rice-crab culture systems of

7	northeast China. Aquacult. Fish. 2019; 4(4):134-141. DOI: 10.1016/j.aaf.2018.12.006.

448 15. Chakravorty A, Ghosh P. Interrelationship and cause effect analysis among panicle yield attributing t

9	raits in lowland Traditional Rice. J of control Europe an Agriculture. 2014; 15(4):213-224. https://hr**c**

50	ak.srce.hr/130764

451 16. Xu M, Ma XZ, Wang W. Effects of different cultivation patterns on rice yield and crab in rice-crab

culture system. Sci Agric Sin. 2014; 47(9):1828-1835. http://en.cnki.com.cn/Article_en/CJFDTotal-Z**N**

53	YK201409018.htm

454 17. Wang H, Zhang CH, Chen JJ, Yan Z, Zhou GX, Huang YL. Research advances in the influence fac

55	tors and related genes of rice quality traits. China Rice. 2018; 24(4):16-21. http://en.cnki.com.cn/Arti**c**

26

6	le_en/CJFDTotal-DAOM201804005.htm

457 18. Li XD, Dong SL, Lei YZ, Li YH. The effect of stocking density of Chinese mitten crab Eriocheir

58	sinensis on rice and crab seed yields in rice–crab culture systems. Aquaculture. 2007; 273:487–493.

459	https://doi.org/10.1016/j.aquaculture.2007.10.028

460 19. Chen XY, Yu FQ, Li ZQ, Sun FY. Comparative analysis of field production and ecological characteristics of

61	common paddy fields and rice crabs. Liaoning Agric Sci. 2017; 2017(2):53-54.

462 20. Hou YP, Han LG, Kong LL,Yin CX, Qin YB, Li Q, et al. Nutrient absorption, translocation in rice463	and soil nitrogen equilibrium under different nitrogen application doses. J Plant Nutr Fert. 2015; 21

(4):836-845. http://en.cnki.com.cn/Article_en/CJFDTOTAL-ZWYF201504002.htm

465 21. Jia D, Lu JJ, Sun YJ, Song S, Du H, Han L. Effect of different nitrogen fertilizer application strategies on rice466	growth and yield. ASAR. 2016; 8(1):33-39. DOI: CNKI:SUN:AAGR.0.2016-01-010.

467 22. Ding WC, Xu XP, He P, Ullah S, Zhang JJ, Cui ZL, et al. Improving yield and nitrogen use effici468	ency through alternative fertilization options for rice in China: A meta-analysis. Field Crop Res. 201

469	8; 227:11-18. https://doi.org/10.1016/j.fcr.2018.08.001

470 23. Kramer AW, Doane TA, Horwath WR, Van Kessel C. Combining fertilizer and organic inputs to syn471	chronize N supply in alternative cropping systems in California. Agr Ecosyst Environ. 2002; 91:233-

243. DOI:10.1016/S0167-8809(01)00226-2.

73 24. An H, Liu MD, Wang HX, Hao XD, Wang YJ. Effects of different rice-crab production models on474	rice-crab yield and quality. Acta Agric Nucl Sin. 2012; 26(3):5081–0586. http://www.cnki.com.cn/Arti

475	cle/CJFDTotal-HNXB201203030.htm

476 25. Walia MK, Walia SS, Sharma S, Dhaliwal SS. Effect of integrated nutrient management approach on

77	nutritional quality of rice grain. Jour Pl Sci Res. 2014; 30(1):33-38.

478 26. Lisle AJ, Martin M, Fitzgerald MA. Chalky and translucent rice grains differ in starch composition

and structure and cooking properties. Cereal Chem. 2000; 77(5):627–632. https://doi.org/10.1094/CC**H**

480	EM.2000.77.5.627

481 27. Xi M, Lin ZM, Zhang XC, Liu ZH, Li GH, Wang QS, et al. Endosperm structure of white-belly a

nd white-core rice grains shown by scanning electron microscopy. Plant Prod Sci. 2015; 17(4):285-**2**

483	90. https://doi.org/10.1626/pps.17.285

484 28. Cheng C, Ali A, Shi QH, Zeng YH, Tan XM, Shang QY, et al. Response of chalkiness in high-qua485	lity rice (Oryza sativa L.) to temperature across different ecological regions. J Cereal Sci. 2019; 87:

86	39-45. https://doi.org/10.1016/j.jcs.2019.02.009

487 29. Deng F, Wang L, Pu SL, Mei XF, Li SX, Li QP, et al. Shading stress increases chalkiness by postponing488	caryopsis development and disturbing starch characteristics of rice grains. Agr Forest Meteorol. 2018;

263:49-58. https://doi.org/10.1016/j.agrformet.2018.08.006

490 30. Zhou LJ, Liang SS, Ponce K, Marundon S, Ye GY, Zhao XQ. Factors affecting head rice yield and chalkiness

91	in indica rice. Field Crop Res. 2015; 172:1-10. https://doi.org/10.1016/j.fcr.2014.12.004

492 31. Qiao JF, Liu ZH, Deng SY, Ning HF, Yang XY, Lin ZM, et al. Occurrence of perfect and imperfec

t grains of six japonica rice cultivars as affected by nitrogen fertilization. Plant Soil. 2011; 349:191**-**

94	202. https://link.springer.com/article/10.1007/s11104-011-0861-4

495 32. Zhu DW, Zhang HC, Guo BW, Xu K, Dai QG, Wei HY, et al. Effects of nitrogen level on yield a496	nd quality of japonica soft super rice. J Integr. Agr. 2017; 16(5):1018-1027. https://doi.org/10.1016/S

497	2095-3119(16)61577-0

498 33. Asghar S, Anjum FM, Amir RM, Khan MA. Cooking and eating characteristics of rice (Oryza sativa L.)-A499	review. Pak. J Food Sci. 2012; 22(3):128-132.

27

500 34. Wei HY, Chen ZF, Xing ZP, Zhou L, Liu QY, Zhang ZZ, et al. Effects of slow or controlled releas501	e fertilizer types and fertilization modes on yield and quality of rice. J Integr. Agr. 2018; 17(10):22

22–2234. https://doi.org/10.1016/S2095-3119(18)62052-0

35. Martin M, Fitzgerald MA. Proteins in rice grains influence cooking properties! J Cereal Sci. 2002; 36:

285-294. https://doi.org/10.1006/jcrs.2001.0465

505 36. Baxter G, Blanchard C, Zhao J. Effects of glutelin and globulin on the physicochemical properties of rice

starch and flour. J Cereal Sci. 2014; 60:414-420. https://doi.org/10.1016/j.jcs.2014.05.002

507 37. Jin ZX, Qiu TQ, Sun YL, Zhao JM, Jin XY. Effect of nitrogen fertilizer on chalkiness ratio and co

8	oking and eating quality properties of rice grain. Plant Nutr Fert Sci. 2001; 7(1):31-35. http://www.**w**

9	anfangdata.com.cn/details/detail.do?_type=perio&amp;id=zwyyyflxb200101005

510 38. Zeng YH, Tan XM, Zeng YJ, Xie XB, Pan XH, Shi QH, et al. Changes in the rice grain quality of different511	high-quality rice varieties released in southern China from 2007 to 2017. J Cereal Sci. 2019;

87:111-116.https://doi.org/10.1016/j.jcs.2019.03.015

513 39. Li HY, Gilbert RG. Starch molecular structure: The basis for an improved understanding of cooked rice

texture. Carbohyd Polym. 2018; 195:9-17. https://doi.org/10.1016/j.carbpol.2018.04.065

515 40. Li HY, Prakash S, Nicholson TM, Fitzgerald MA, Gilbert RG. The importance of amylose and amyl516	opectin fine structure for textural properties of cooked rice grains. Food Chem. 2016; 196:702-711.

7	https://doi.org/10.1016/j.foodchem.2015.09.112

518 41. Li HY, Fitzgerald MA, Prakash S, Nicholson TM, Gilbert RG. The molecular structural features cont519	rolling stickiness in cooked rice, a major palatability determinant. Sci Rep. 2017a; 7, 43713. https://

www.ncbi.nlm.nih.gov/pubmed/21480275

Click here to access/download;Figure;Fig 1.tif.tif

Click here to access/download;Figure;Fig 2.tif.tif

Click here to access/download;Figure;Fig 3.tif.tif

Click here to access/download;Figure;Fig 4.tif.tif
