# Supplementary material for: Effects of different fertilization modes on rice yield and quality under a rice-crab culture system
Source: PLoS One. 2020 Mar 25;15(3):e0230600. doi: 10.1371/journal.pone.0230600 (PMC7094854; doi:10.1371/journal.pone.0230600)
Supplement: Attachment — Submitted filename: Response to Reviewers.docx [file pone.0230600.s002.docx]

Response to Reviewers

Dear Editor：

We sincerely thank the reviewers for their insightful comments. Our response to their feedback is given point-by-point below.

1. Is the manuscript technically sound, and do the data support the conclusions?

The manuscript must describe a technically sound piece of scientific research with data that supports the conclusions. Experiments must have been conducted rigorously, with appropriate controls, replication, and sample sizes. The conclusions must be drawn appropriately based on the data presented.

Reviewer #1: Yes

Responses to Questions:

The manuscript describes a scientifically sound study. This experiment investigated the effects of three different fertilization modes on rice yield and quality in a rice-crab culture system in Panjin, China. All indicators were tested in accordance with national standards or recognized scientific methods. The study was conducted in the field using a randomized complete block design with three replicates, including three non-fertilized control groups, for a total of 12 rice samples. Special personnel were responsible for the rice planting, fertilization, and harvesting processes, and the operation was rigorous and standard. The conclusions were based on statistical analysis of the data, and are consistent with the results of the one-year preliminary experiment.

2. Has the statistical analysis been performed appropriately and rigorously? 

Reviewer #1: Yes

Responses to Questions:

The data measured in this test were collated by Excel 2010, and the average and standard deviation of three repeated tests for each index were calculated. SPSS 25 software was used to perform a one-way ANOVA test using the LSD method and Duncan method, with a significance level of 0.05, and a Pearson correlation coefficient was used for bivariate correlation analysis.

3. Have the authors made all data underlying the findings in their manuscript fully available?

The [PLOS Data policy](http://www.plosone.org/static/policies.action#sharing) requires authors to make all data underlying the findings described in their manuscript fully available without restriction, with rare exception (please refer to the Data Availability Statement in the manuscript PDF file). The data should be provided as part of the manuscript or its supporting information, or deposited to a public repository. For example, in addition to summary statistics, the data points behind means, medians and variance measures should be available. If there are restrictions on publicly sharing data—e.g. participant privacy or use of data from a third party—those must be specified.

Reviewer #1: Yes

Responses to Questions:

In our analysis, we have made full use of all the data in the manuscript to reach the most accurate and reliable conclusions. All the data on which the figures in the manuscript are based, including the mean, standard deviation, and significant differences, are stored in a public repository recommended by PLOS– figshare. The data involved are data for all indicators of rice cooking and eating, and nutritional quality. The data listed in the tables are real data.

4. Is the manuscript presented in an intelligible fashion and written in standard English?

   PLOS ONE does not copyedit accepted manuscripts, so the language in submitted articles must be clear, correct, and unambiguous. Any typographical or grammatical errors should be corrected at revision, so please note any specific errors here.

   Reviewer #1: Yes

Responses to Questions:

The manuscript has been written in standard English. The author has corrected the grammatical errors pointed out by the reviewers and has carefully checked them.

5. Review Comments to the Author

Please use the space provided to explain your answers to the questions above. You may also include additional comments for the author, including concerns about dual publication, research ethics, or publication ethics. (Please upload your review as an attachment if it exceeds 20,000 characters)

Reviewer #1: plz consider the minor corrections have been inserted in ur manuscript in the attach file.
plz try to rewrite the abstract more clear than the current one, since it is has to all treatment.
plz see the attach file

Responses to Questions:

Thank you very much for the excellent suggestions listed in our manuscript. We confirm that this work has not been published elsewhere and that all listed authors have approved the manuscript. All rice samples used in the test were taken from the rice experimental demonstration base of the national R&D project. Therefore, this manuscript will not encounter issues such as dual publishing and contravention of research ethics.

We are very grateful to the reviewers for their valuable comments. The author has adopted and revised the text according to the feedback and made appropriate supplementary amendments to the manuscript.

In response to your questions about the proportion of organic and inorganic fertilizers in the FP2 fertilization mode, we added an explanation in the Materials and Methods section as follows. “The ratio of inorganic nitrogen to organic nitrogen was 7:3, inorganic nitrogen provided 70% of the nitrogen in the mixed fertilizer and organic nitrogen provided 30% of the nitrogen. Chicken manure was used as organic fertilizer, and the nitrogen content was determined in advance.”

Regarding your questions about high available nitrogen and low available potassium, we offer the following explanation. The Panjin area has coastal saline-alkali soil. We have tested the available nitrogen content in the soil at 85–120 mg/kg for many years. The effective nitrogen content determined by this test was 105.2 mg/kg, which is normal. Regarding potassium, our test results were indeed low, which may be related to the soil conditions of the test field.

Finally, we modified the abstract and deleted the redundant description of the three different fertilization modes because their specific descriptions are included in the materials section, which makes the abstract clearer and more concise.

6. PLOS authors have the option to publish the peer review history of their article ([what does this mean?](https://journals.plos.org/plosone/s/editorial-and-peer-review-process#loc-peer-review-history)). If published, this will include your full peer review and any attached files.

   If you choose “no”, your identity will remain anonymous but your review may still be made public.

   **Do you want your identity to be public for this peer review?** For information about this choice, including consent withdrawal, please see our [Privacy Policy](https://www.plos.org/privacy-policy).

Reviewer #1: No

Responses to Questions:

Please do not reveal my identity for this peer review. I would prefer to remain anonymous. Thank you.

Thank you again for your time! If some parts of the manuscript still need to be modified, we look forward to further guidance from the reviewers.

Yours sincerely

Zhaoxia Wu
